# The 3D ultrastructure of the chordotonal organs in the antenna of a microwasp remains complex although simplified

**DOI:** 10.1038/s41598-022-24390-4

**Published:** 2022-11-23

**Authors:** Anna V. Diakova, Anastasia A. Makarova, Song Pang, C. Shan Xu, Harald Hess, Alexey A. Polilov

**Affiliations:** 1 Department of Entomology, Faculty of Biology, Lomonosov Moscow State University, Moscow, Russia; 2 Janelia Research Campus of the Howard Hughes Medical Institute, Ashburn, USA; 3 Yale School of Medicine, New Haven, CT USA; 4 Department of Cellular and Molecular Physiology, Yale School of Medicine, New Haven, CT USA

**Keywords:** Zoology, Entomology

## Abstract

Insect antennae are astonishingly versatile and have multiple sensory modalities. Audition, detection of airflow, and graviception are combined in the antennal chordotonal organs. The miniaturization of these complex multisensory organs has never been investigated. Here we present a comprehensive study of the structure and scaling of the antennal chordotonal organs of the extremely miniaturized parasitoid wasp *Megaphragma viggianii* based on 3D electron microscopy. Johnston’s organ of *M. viggianii* consists of 19 amphinematic scolopidia (95 cells); the central organ consists of five scolopidia (20 cells). Plesiomorphic composition includes one accessory cell per scolopidium, but in *M. viggianii* this ratio is only 0.3. Scolopale rods in Johnston’s organ have a unique structure. Allometric analyses demonstrate the effects of scaling on the antennal chordotonal organs in insects. Our results not only shed light on the universal principles of miniaturization of sense organs, but also provide context for future interpretation of the *M. viggianii* connectome.

## Introduction

In many animals, multiple senses can be performed by one organ, which then acquires sophisticated structure and combines receptors responding to a variety of modalities. Of all sense organs studied, insect antenna is perhaps the most versatile, combining four Aristotelian senses (olfaction, taste, mechanoreception, audition)^1,2^. An important part of antennal sensory system are chordotonal organs responsible for sensing antennal vibrations: Johnston’s organ and the central organ. Johnston’s organ has multiple functions: perception of air or water current and electric fields^3^, flight control^4^, audition, and graviception^2^. Chordotonal organs consist of sensory units, scolopidia, which attach to the next segment either by a cap (mononematic scolopidia) or a tube (amphinematic scolopidia). Typically, Johnston’s organ scolopidia are amphinematic and contain three sensory neurons enveloped by three cells: accessory cell, scolopale cell, and attachment cell. It has up to 7500 scolopidia/35 000 cells^5^ and is found in nearly all insects. Mononematic scolopidia of the central organ usually consist of two neurons, scolopale cell and attachment cell. The central organ has up to 50 scolopidia/200 cells^6^ and is not always present.

Morphological studies on Hymenoptera, however, are scarce, though many insects were studied thoroughly^5,7^. Several studies aim to reconstruct and analyze auditory neural pathways of Johnston’s organ^8,9^. A number of studies explore auditory mechanisms, uncovering a process of signal transduction and sound amplification remarkably similar to those at work in vertebrate ears^10–13^. Moreover, studies on *Drosophila* auditory associated genes revealed that 1/5 of them have human homologs that are involved in hearing disorders^14,15^. These striking similarities to vertebrate ears make insects a suitable object for research on the fundamentals of hearing.

Still, there is no data on miniaturization of chordotonal organs in insects smaller than some unicellular organisms, such as egg-parasitoid wasps of the genus *Megaphragma*. Their body size is only 200 µm, and their organs, cells, and even organelles possess numerous size-related adaptations^16,17^. In our previous study, we found that the number of antennal sensilla is dramatically reduced in *Megaphragma*, but miniaturization had little to no effect on the number of antennal sensilla types and sizes of the sensilla, and sensillum morphology did not change dramatically^18^. *Megaphragma* has complex and diverse behavior, indicating high level of functionality of its antennae. In this work, we explore the optimization of the sophisticated structure of antennal chordotonal organs in extremely miniaturized *Megaphragma viggianii* (Hymenoptera: Trichogrammatidae).

## Results and discussion

**Figure 1:**
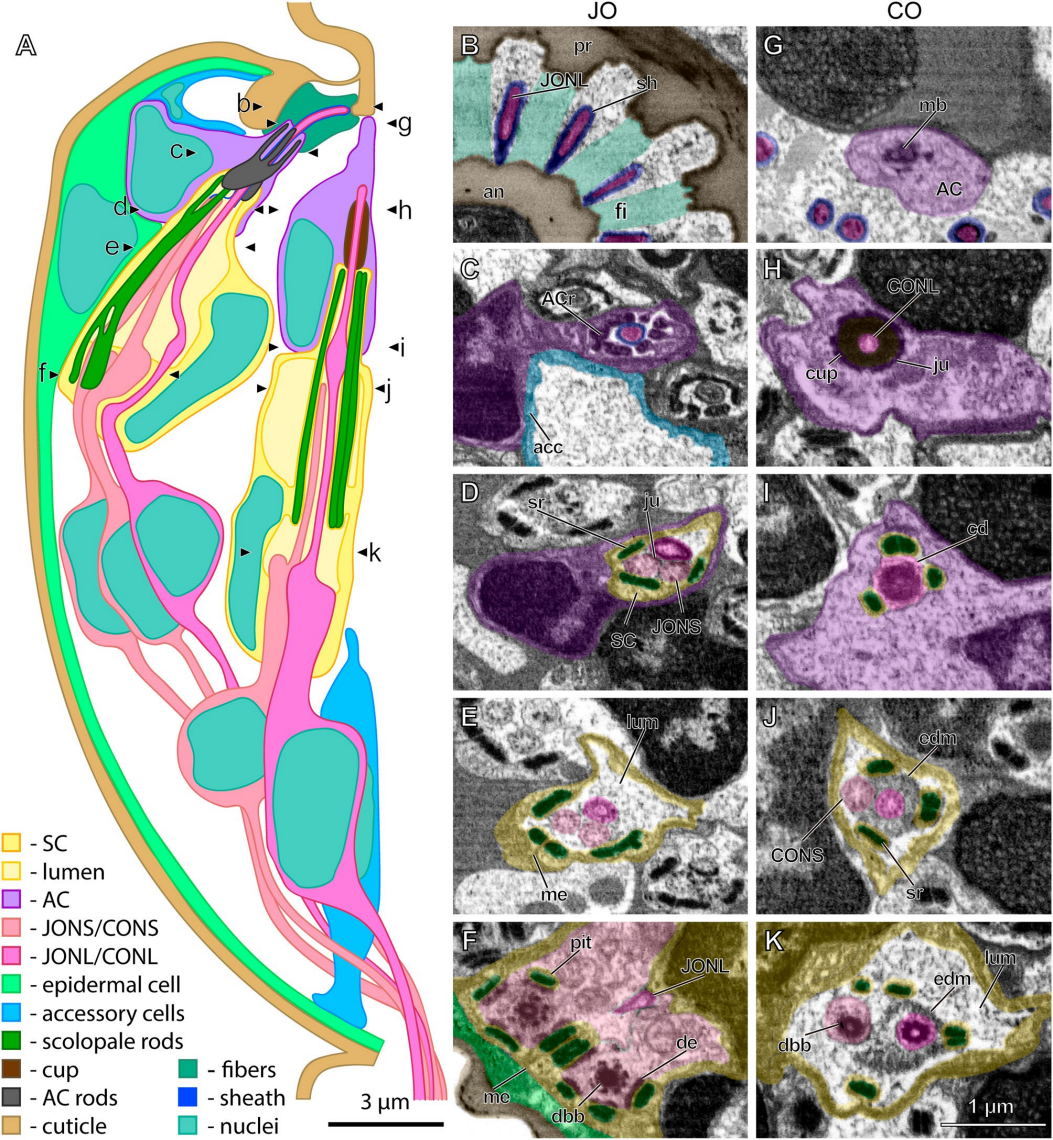
Ultrastructure of pedicellar chordotonal organs in *Megaphragma viggianii*. (**A**) Scheme of longitudinal section of *M. viggianii* pedicel with one Johnston’s organ (JO) and one central organ (CO) scolopidia. Letters designate the approximate location of cross sections shown in B–K. (B–F): Cross sections of JO scolopidium (FIB-SEM). (**B**) A sheath covers the tips of long JO neurons (JONL) cilia that lie between the fibers connecting pedicel rim and annelus. (**C**) Attachment cell (AC) forms a thin channel containing JONL cilium tip. Accessory cell surrounds a cavity filled with granular material. (**D**) Projections of scolopale cell (SC) containing scolopale rods surround JO cilia tips connected with junctions. (**E**) JO cilia lie in a lumen surrounded by SC that forms mesaxon. (**F**) Bulbs of short JO neurons (JONS) dendrites are joined by desmosomes to the scolopale rods, one rod is inserted in a small pit. (**G**–**K**): Cross sections of CO scolopidium (FIB-SEM). (**G**) A bundle of microtubules is observed in AC. (**H**) Tip of long CO neuron (CONL) cilium fits inside of a cap joined to the AC by junctions. (**I**) Four scolopale rods form a triangle around the cilium dilation of CONL. (**J**) A band of electron-dense material lies below the cilium dilation. (**K**) Distal basal bodies lie in CONL and short CO neuron (CONS) Cilia surrounded by electron-dense material. (acc—accessory cell, ACr—AC rods, an—annelus, cd—cilium dilation, dbb—distal basal body, de—desmosome, edm—electron-dense material, fi—fiber, ju—junction, lum—lumen, mb—microtubule bundle, me—mesaxon, pr—pedicel rim, sh—sheath, sr—scolopale rod).

**Figure 2:**
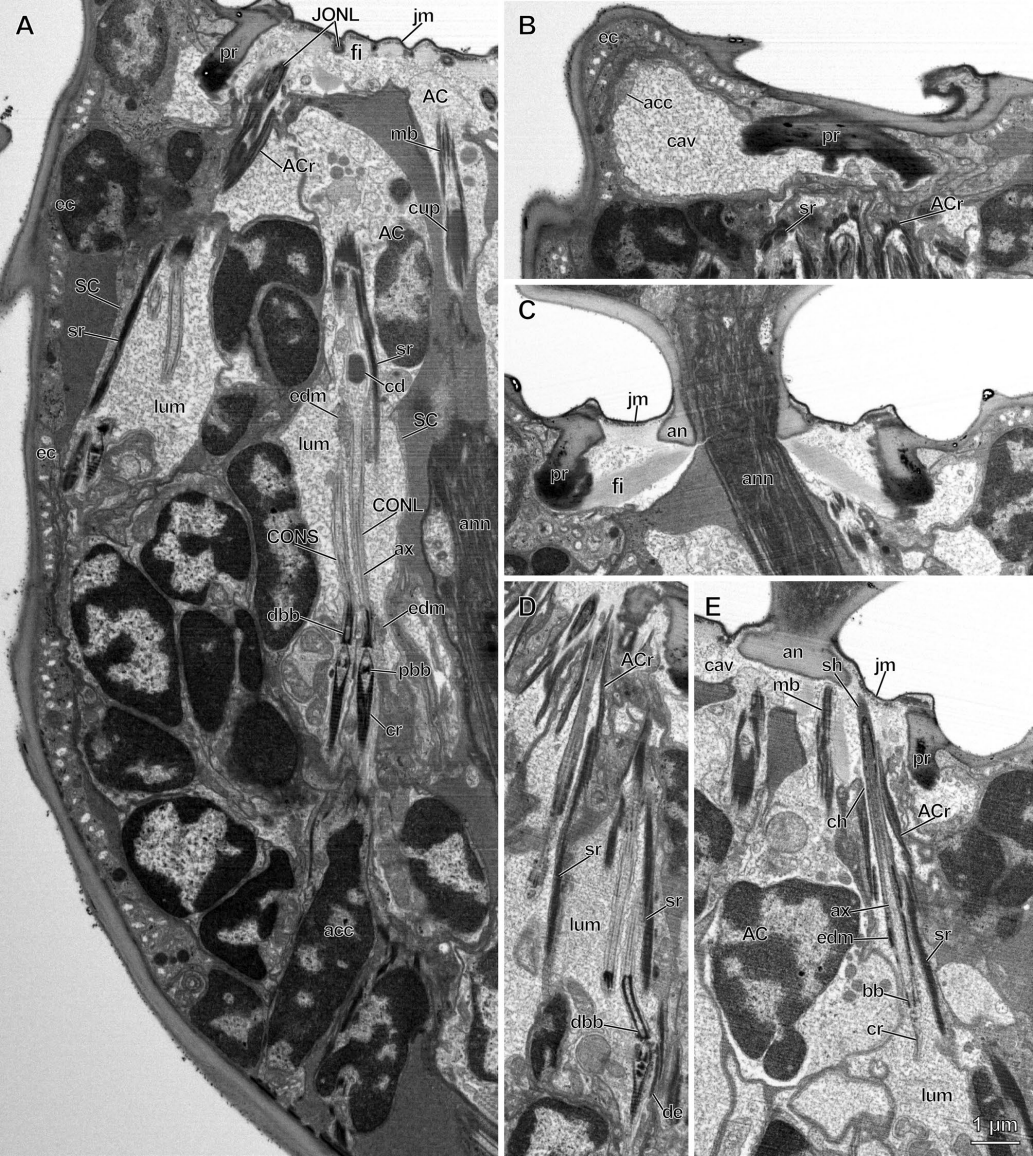
Longitudinal sections of pedicellar chordotonal organs in *Megaphragma viggianii*. (**A**) Median section of pedicel, showing the dorsal scolopidia. Basal bodies, cilia roots and axonemes are visible in central organ (CO) cilia which are surrounded by two bands of electron-dense material. Cilium dilation of long CO neuron (CONL) encloses a core. Scolopale cell (SC) form lumina, scolopale rods and cups. Attachment cells (AC) of Johnston’s organ (JO) form AC rods, and AC of CO form microtubule bundles. Cilia tips of long JO neurons (JONL) run alongside the invaginations of joint membrane between fibers. (**B**) Parasagittal section of the distal part of the pedicel. Accessory cell forms cavity filled with granular material. (**C**) Median section of the distal part of the pedicel. A joint membrane and fibers connect the pedicel rim and annelus. (**D**) Longitudinal section through the distal part of ventral JO scolopidia. A distinct bend of short JO neuron (JONS) cilium is observed. (**E**) Parasagittal section through the distal part of the pedicel. JONL contains only one basal body, a small ciliary root and an axoneme distally tapering in a bundle of microtubules. Its sheath-covered tip attaches to the annelus alongside CO AC. A narrow channel formed by the JO AC connects the lumen and cavity. (acc—accessory cell, ACr—AC rods, an—annelus, ann—antennal nerve, ax—axoneme, bb—basal body, cav—cavity, cd—cilium dilation, ch—channel, cr—cilia root, dbb—distal basal body, de—desmosome, ec—epidermal cell, edm—electron-dense material, fi—fiber, jm—joint membrane, ju—junction, lum—lumen, mb—microtubule bundle, me—mesaxon, pbb—proximal basal body, pr—pedicel rim, sh—sheath, sr—scolopale rod).

**Figure 3:**
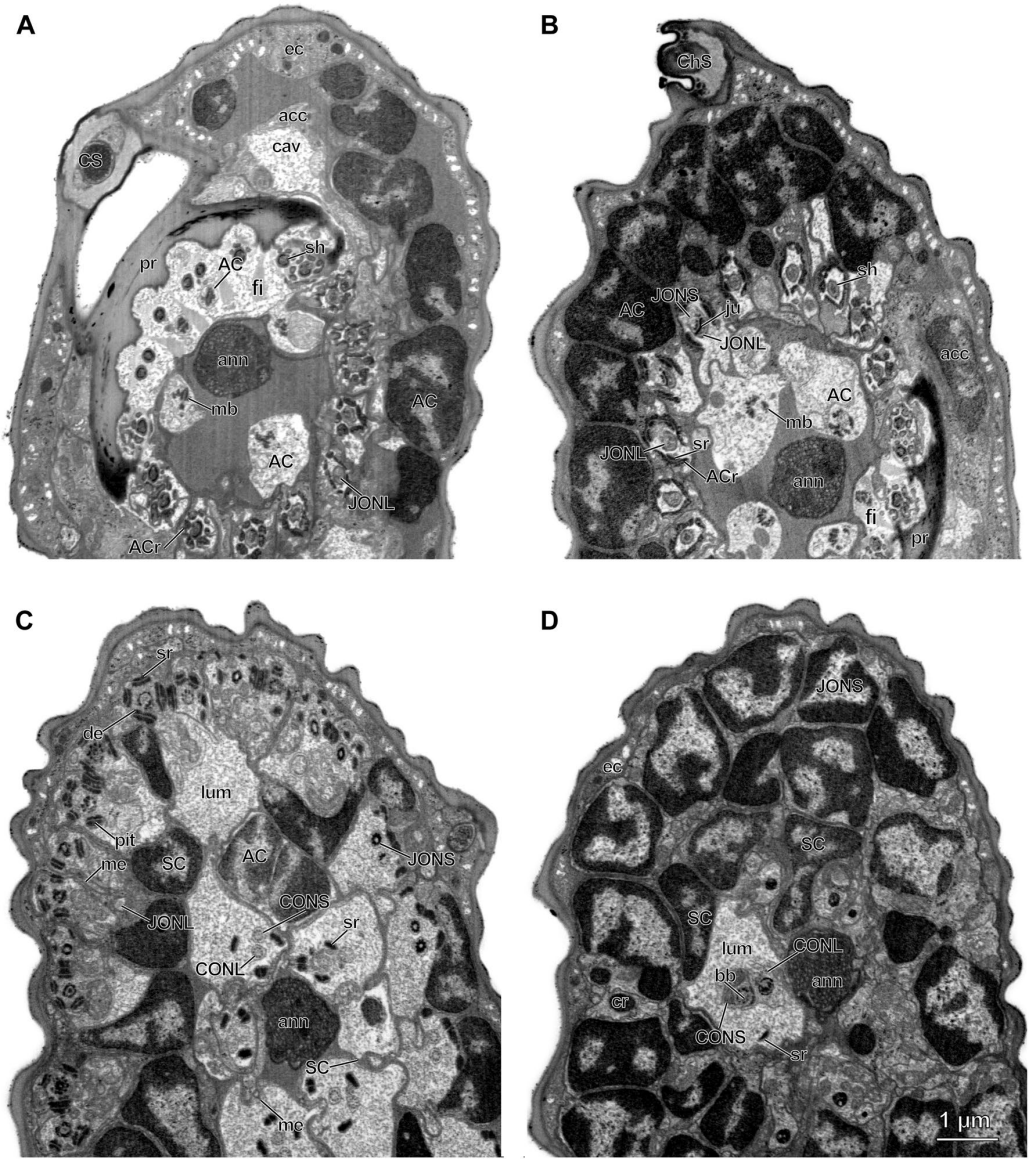
Cross sections of *Megaphragma viggianii* pedicellar chordotonal organs. (**A**) Section through the distal end of the pedicel. Antennal nerve is surrounded by five central organ (CO) attachment cells (AC) and tips of long Johnston’s organ neurons (JONL) cilia. Nuclei of Johnston’s organ (JO) AC occupy the outermost part of the pedicel, adjoining to the epidermal and accessory cells. A sensillum campaniformium (CS) is observed in the periphery. (**B**) Cross section at the 4/5 of the pedicel length. Cell bodies of JO AC are tightly packed, inside their rods lie the tips of scolopale rods. An intercellular junction between JO cilia is visible. The base of the sensillum chaeticum is observed in the periphery. (**C**) Cross section at the 2/3 of the pedicel length. Dendrites of short JO neurons (JONS) lie in the periphery; desmosomes connect them to the scolopale rods. Large lumina are formed by JO scolopale cells (SC), whose nuclei acquire twisted shapes to provide space for it. (**D**) Section through the middle of the pedicel. Neuron bodies surround CO scolopidia and antennal nerve, they are tightly packed. Elongated and twisted SC nuclei occupy the outermost sides of CO scolopidia. (acc—accessory cell, ACr—AC rods, an—annelus, ann—antennal nerve, ax—axoneme, bb—basal body, cav—cavity, cd—cilium dilation, ch—channel, ChS—sensillum chaeticum, CS—sensillum campaniformium, cr—cilia root, dbb—distal basal body, de—desmosome, ec—epidermal cell, edm—electron-dense material, fi—fiber, jm—joint membrane, ju—junction, lum—lumen, mb—microtubule bundle, me—mesaxon, pbb—proximal basal body, pr—pedicel rim, sh—sheath, sr—scolopale rod).

In female *M. viggianii* the second antennomere, pedicel, is barrel-shaped, 30 µm in length and 20 µm in diameter (Fig. 1). Distally pedicel forms a circular apodeme with 14 electron-dense prongs. A sub-segment, anellus, is inserted in the distal end of the pedicel. It forms a narrow cuticular cylinder with a circular rim at the end bearing 14 small protrusions. 14 cuticle fibers about 0.3 µm wide connect each of the pedicel prongs and anellus protrusions (Fig. 2C). They lie in a cavity formed by two accessory cells, filled with granular material (Figs. 2B, 3A). Half of them, three fibers in dorsal-lateral quarter and four fibers in ventral-medial quarter, are considerably elongated proximally. The joint is covered with a thin layer of membranous cuticle (60–70 nm) (Fig. 2A). There are only four epidermal cells, their highly vacuolated cytoplasm is flattened out, covering cuticle on the inside (Videos 1, 2,3).

**Figure 4:**
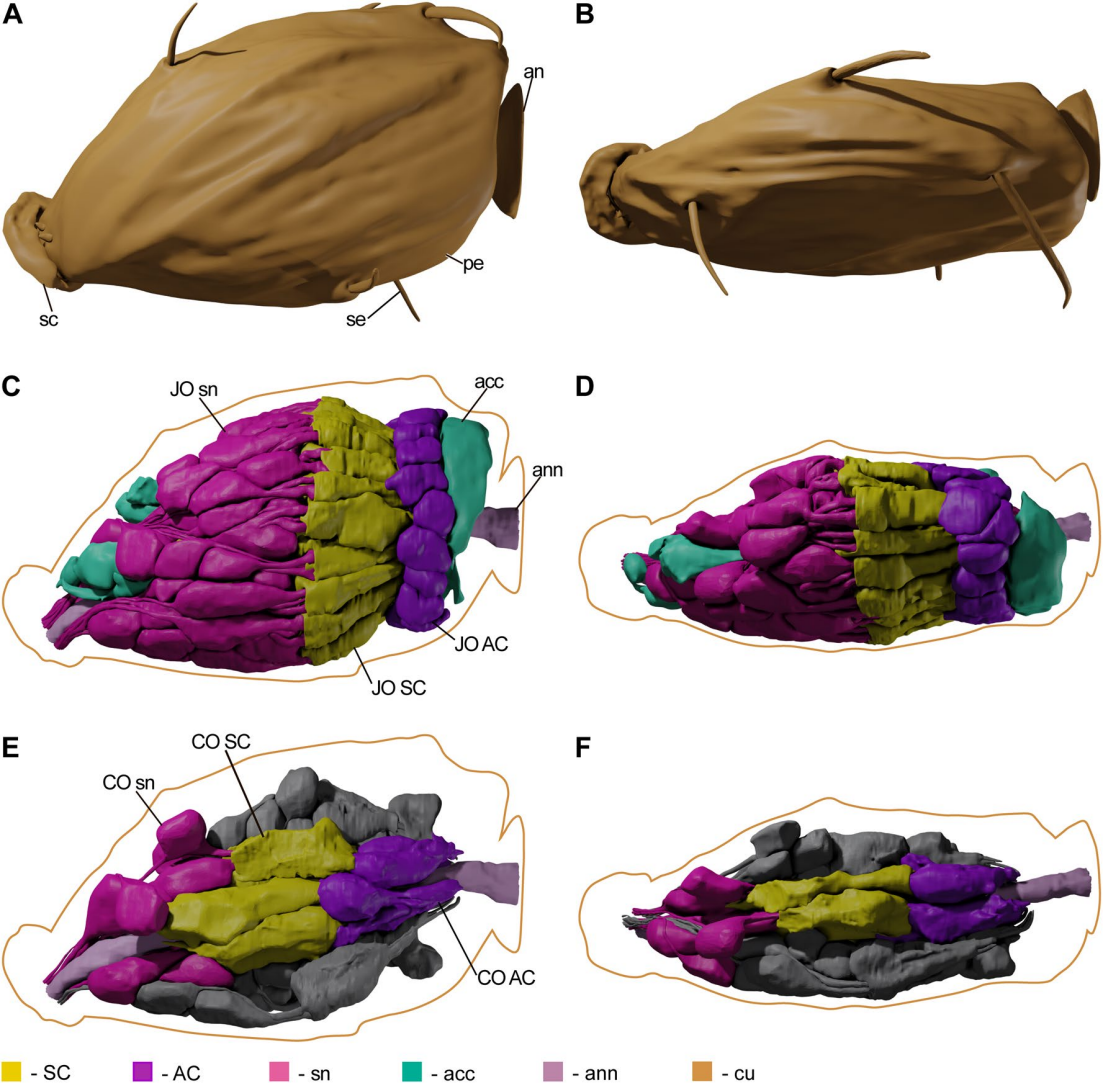
3D models of *M. viggianii* pedicel, JO and CO. (**A**), (**C**), (**E**)—medial view, (**B**), (**D**), (**F**)—dorsal view. (**A**), (**B**): cuticular surfaces; (**C**), (**D**): JO scolopidia and accessory cells; (**E**) (**F**): CO scolopidia, half of the JO scolopidia models are absent; the present half is colored grey. (acc—accessory cell, an—annelus, ann—antennal nerve, cu—cuticle, pe—pedicel, sc—scape, se—sensilla, sn—sensory neurons).

19 JO scolopidia are arranged circularly, occupying the periphery of the pedicel. They are amphinematic, 15.5 ± 0.87 µm in length and 3.7 ± 1.0 µm in width (Video 1Fig. 4). Each scolopidium contains 3 neurons, a scolopale cell (SC) and an attachment cell (AC) (Video 4). Sensory neurons are bipolar, with bodies situated proximally. They each bear a dendrite consisting of an inner dendritic segment (IDS) and an outer dendritic segment—a modified cilium with a 9 × 2 + 0 axoneme structure. Two of the neurons (JONS—JO Neuron Short) in each scolopidium have shorter dendrites with IDS 4.4 ± 0.87 µm long and 0.84 ± 0.22 µm wide, and cilia 6.5 ± 0.38 µm long of a uniform diameter throughout (Table 1).

**Table 1:** Dimensions of cells and cellular structures of female *Megaphragma viggianii* chordotonal organs (Johnston’s organ (JO) and central organ (CO)); mean ± SD (n), µm. JONL—long JO neurons, JONS—short JO neurons, CONS—short CO neurons, CONL—long CO neurons, IDS—inner dendritic segment.

Structure	Length	Diameter
JO scolopidium	15.6 ± 0.87 (10)	3.7 ± 1.0 (10)
CO scolopidium	21.8 ± 1.43 (6)	3.6 ± 0.59 (6)
JO scolopale rod	8.1 ± 0.23 (10)	0.38 ± 0.12 (10)
CO scolopale rod	8.3 ± 0.83 (10)	0.27 ± 0.06 (10)
JONL IDS	8.9 ± 0.65 (10)	0.36 ± 0.05 (10)
JONS IDS	4.4 ± 0.87 (10)	0.84 ± 0.22 (10)
JONL cilium	6.8 ± 0.39 (10)	0.31 ± 0.02 (10)
JONS cilium	6.5 ± 0.38 (10)	0.30 ± 0.02 (10)
CONL IDS	4.9 ± 1.65 (6)	0.69 ± 0.10 (6)
CONS IDS	4.5 ± 0.93 (6)	0.47 ± 0.09 (6)
CONL cilium	10.4 ± 0.69 (6)	0.30 ± 0.02 (10)
CONS cilium	5.9 ± 0.49 (6)	0.30 ± 0.02 (10)
Cup	2.0 ± 0.2 (10)	0.65 ± 0.06 (10)
Cilium dilation	–	0.59 ± 0.05 (10)

Cilia in both JO and CO are of the same diameter, 0.3 ± 0.02 µm. IDS form mitochondria-filled bulbs just below cilial segment. Two basal bodies lie in the base of the axoneme, distal basal body connected to the thick and long ciliary roots that surround proximal basal body. The distal body is often tilted in central direction, leading to the bending of cilium (Fig. 2D). Third neuron (JONL—JO Neuron Long) has a longer sensory process with IDS 8.9 ± 0.65 µm long and cilium 6.8 ± 0.39 µm long (Tables 1, S1). Its IDS is very thin (0.36 ± 0.05 µm) and twisted, with a small dilation just before the cilium encompassing a thin and short cilia root. Its cilium has only one short basal body and its axoneme distally transforms in a bundle of microtubules (Figs. 1G, 2E). Tips of JONS cilia are connected with each other and JONL cilium via intercellular junctions (Figs. 1D, 3B). JONL cilium protrudes further, and a thin tubular sheath covers its tip (Fig. 2E). A discontinuous electron-dense layer lies between the sheath and microtubules (Figs. 1D, 2E). JONL tip runs between the cuticle fibers, alongside the joint membrane, which forms depressions above each tip (Fig. 3A). Sometimes a thin layer of electron-dense material between the joint membrane and JONL cilia is observed in depressions. JONL cilium tip inserts in a small indentation on the circular rim of the anellus. 10 JONL cilia are paired in 5 indentations, and 9 indentations contain 1 cilium each.

Neuron cell bodies are 12.5 ± 1.3 µm^3^ in volume in JONS and 8.9 ± 0.62 µm^3^ in volume in JONL (Table 2; raw data—Table S9). They lie proximally, with JONL body often being the most proximal in scolopidium. Large nuclei occupy 0.73 ± 0.06 of cell body volume in JO neurons. JONS have the largest nuclei in JO (7.9 ± 0.55 µm^3^). They also have the most numerous (5.0 ± 1.69) and largest (0.78 ± 0.23 µm^3^) mitochondria, which are situated not only in the cell body, but also in dendrites and dendritic bulbs. JONS mitochondria are of various shapes, round or elongated, a few are large and arborized. JONL have nuclei of 6.8 ± 0.74 µm^3^ and 2.5 ± 0.58 small, elongated mitochondria lying around nucleus or rarely in dendrite, 0.11 ± 0.03 µm^3^ in volume (Tables 2, S2, S3).

**Table 2:** Cell and organelle volumes (µm^3^) and number of mitochondria of female *Megaphragma viggianii* chordotonal organs, mean ± SD (n), raw data—Table S8, S9.

Cell type	Cell volume	Nuclei volume	Mitochondria volume	Number of mitochondria
JO neurons	11.3 ± 2.1 (57)	7.5 ± 0.81 (15)	0.56 ± 0.38 (15)	4.8 ± 1.8 (15)
JONL	8.9 ± 0.62 (19)	6.8 ± 0.74 (5)	0.11 ± 0.03 (5)	2.5 ± 0.58 (5)
JONS	12.5 ± 1.3 (38)	7.9 ± 0.55 (10)	0.78 ± 0.23 (10)	5.0 ± 1.7 (10)
JO SC	19. 6 ± 1.8(19)	6.3 ± 0.44 (5)	0.26 ± 0.2 (5)	3.0 ± 2.2 (5)
JO AC	10.3 ± 1.0 (19)	5.9 ± 0.2 (5)	0.090 ± 0.07 (5)	1.5 ± 0.58 (5)
CO neurons	15.7 ± 5.7 (10)	9.0 ± 2.3 (8)	1.5 ± 0.77 (8)	11.0 ± 5.3 (8)
CONL	20.2 ± 4.6 (5)	10.9 ± 1.6 (4)	2.1 ± 0.64 (4)	13.7 ± 6.2 (4)
CONS	11.2 ± 0.70 (5)	7.1 ± 0.10 (4)	0.90 ± 0.22 (4)	8.2 ± 2.6 (4)
CO SC	24.5 ± 5.6 (5)	7.6 ± 0.42 (4)	0.75 ± 0.18 (4)	4.2 ± 1.7 (4)
CO AC	20.2 ± 2.0 (5)	8.2 ± 0.22 (4)	0.31 ± 0.04 (4)	4.2 ± 2.6 (4)

**Figure 5:**
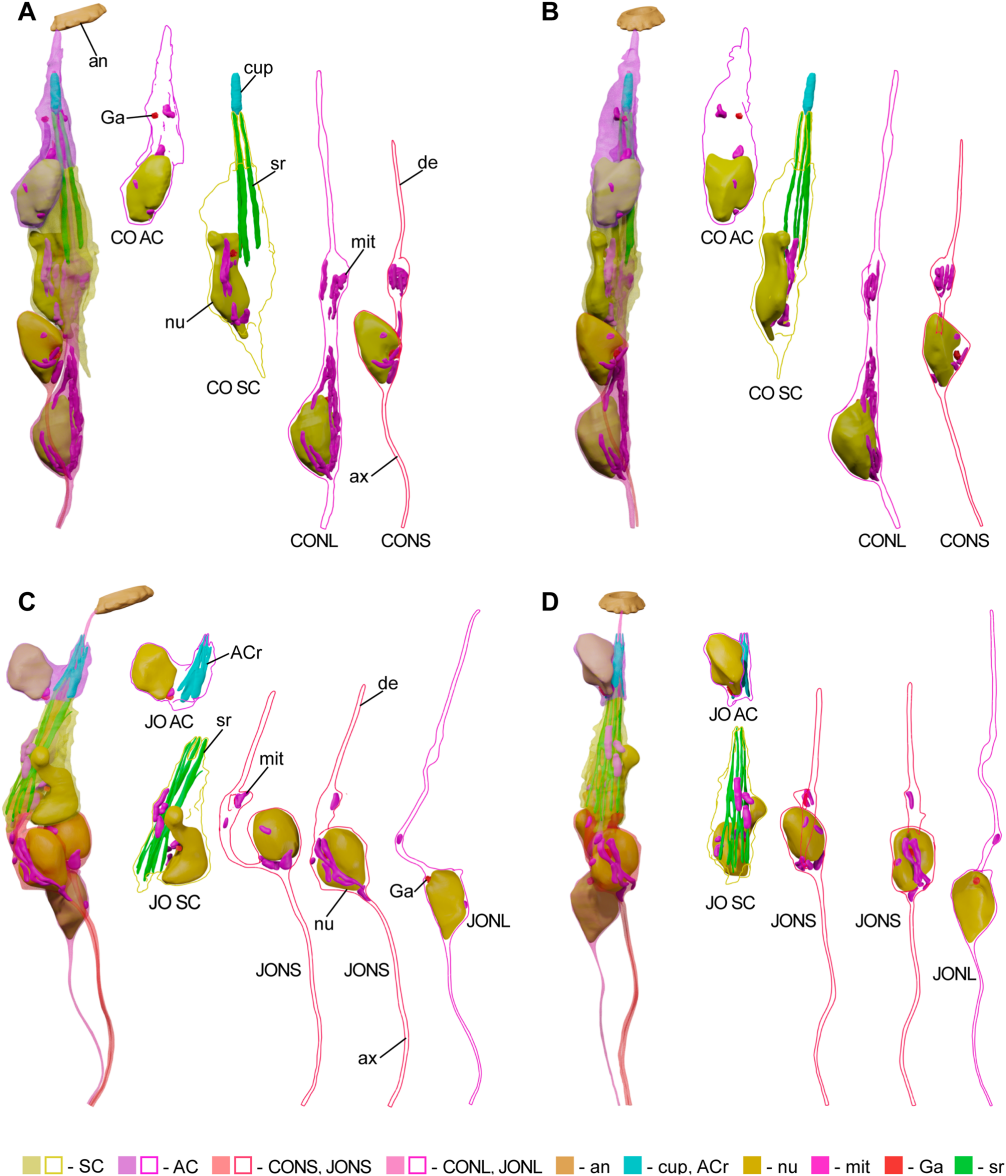
3D models of *M. viggianii* JO and CO cells and their organelles. Each scolopidium is shown with transparent cell surfaces, subsequent images demonstrate each cell with the suface shown as an outline. (**A**), (**B**) CO scolopidium; (**C**), (**D**) JO scolopidium. (ACr—AC rods, an—annelus, ax—axon, de—dendrite, Ga—Golgi apparatus, mit—mitochondria, nu—nucleus, sr—scolopale rods).

SC surrounds distal parts of IDS and cilia, forming a mesaxon and encompassing lumen filled with granular material (Fig. 1F). It produces four intracellular electron-dense scolopale rods arranged in an open mesh structure, which reinforces the periphery of the scolopale lumen (Figs. 2A,D,E, 5, Video 4). Proximally, rods are located only on the outermost side of the scolopidium; distally, the structure curves, but it remains open, so the inner side of the scolopidium is not covered with rods. Rods protrude distally, forming processes of SC. Scolopale rods are 8.1 ± 0.23 µm long and 0.38 ± 0.12 µm wide. IDS of the neurons with shorter dendrites are joined to the adjacent scolopale rods by desmosomes in the bulb region. Some scolopale rods are anchored proximally in small pits in dendritic bulbs (Figs. 1F, 3C).

Cell bodies of SC are 19.6 ± 1.8 µm^3^ in volume; they cover the central parts of scolopidia. Nuclei 6.3 ± 0.44 µm^3^ in volume occupy 0.34 ± 0.03 of cell bodies. Nuclei in dorsal and lateral scolopidia are situated along the inner side of SC, except for one which lies completely on the outermost side. Nuclei in the medial and ventral scolopidia are twisted in the middle, with one half lying in the inner side and the other occupying the outermost side of SC. There are 3.0 ± 2.1 peripheral mitochondria in SC, long and sometimes bent, 0.26 ± 0.20 µm^3^ in volume (Tables 2, S2, S3).

AC encloses the tips of the cilia and SC processes and forms the continuation of the lumen: a narrow channel, which opens in a cavity occupied by ensheathed JONL cilia tips lying between cuticular fibers in granular material. Three wide intracellular rods (Figs. 2A,E, 5, Video 4) support the channel, distally fusing together and then dividing in 6 processes, which surround the tip of JONL cilium.

Nuclei are 5.86 ± 0.20 µm^3^ in volume, they fill 0.53 ± 0.02 of the AC bodies (10.3 ± 1.0 µm^3^ in volume), lying in the outermost side of AC. A few (1.5 ± 0.58) elongated, straight, longitudinally oriented mitochondria 0.090 ± 0.07 µm^3^ in volume are observed proximally (Tables 2, S2, S3).

CO consists of 5 mononematic scolopidia situated circumferentially inside the ring of JO scolopidia, around the antennal nerve (Fig. 4, Video 1). They are significantly longer than JO scolopidia (21.8 ± 1.4 µm) and about the same width (3.6 ± 0.59 µm) (Tables 1, S1). CO scolopidia contain one less sensory neuron than JO—they consist of two neurons, SC and AC (Video 5). There are 4 accessory cells filled with electron-dense granules, attaching CO to the epidermis. Their cell bodies are attached to the epidermal cells by desmosomes, while their projections are wedged in-between SC and neuron bodies (Fig. 2A, Video 5). One spherical accessory cell lies proximally and does not reach the scolopidia. Sensory neurons are of the same constitution as in JO: they are bipolar, with a single dendrite comprised of an IDS and a modified cilium. One of the neurons (CONS—CO Neuron Short) in each scolopidium has a shorter cilium (5.9 ± 0.49 µm). CONS have IDS 4.5 ± 0.93 µm long and 0.47 ± 0.09 µm wide (Table 1). Their cilia have a uniform diameter and a continuous axoneme throughout. In their base lie two basal bodies, proximal basal bodies situated in IDS bulbs containing ciliary roots and mitochondria (Fig. 3D).

Second neuron (CONL—CO Neuron Long) has a longer cilium (10.4 ± 0.69 µm); its IDS is wider than in CONS (0.69 ± 0.1 µm), 4.9 ± 1.65 µm long, with a bulb, that sometimes surrounds the bulb of CONS IDS (Tables1, S1). Both CONS and CONL have long and thick ciliary roots situated in IDS and immersed in cell bodies. At the level of CONS cilium termination, CONL cilium forms a dilation 0.59 ± 0.05 µm in diameter with electron-dense spherical core (Figs. 1H, 2A). Microtubules diverge, surround the core and then revert to the original structure distally. The tip of CONL inserts into an electron-dense extracellular cylindrical cap (Figs. 1H, 2A, Video 5).

CONL have the largest cell body volume along JO and CO neurons (20.2 ± 4.6 µm^3^); CONS cell body is 11.2 ± 0.7 µm^3^ in volume (Tables 2, S2, S3). Nuclei occupy 0.53 ± 0.08 of cell body volume in CO neurons. CONL nuclei are the largest among all cells in JO and CO (10.9 ± 1.5 µm^3^), CONS have nuclei 7.1 ± 0.10 µm^3^ in volume. CO neurons have 11.0 ± 5.3 mitochondria per cell. They are scattered around nucleus, alongside GA, and in IDS, especially in bulbs. CONL have the most voluminous mitochondria in CO (2.1 ± 0.64 µm^3^), while CONS mitochondria are 0.90 ± 0.22 µm^3^ in volume altogether (Tables 2, S2, S3).

SC of CO are larger than those of JO (24.5 ± 5.6 µm^3^) (Tables 2, S2, S3). There are four scolopale rods, in the second specimen two scolopidia with 5 rods are present. CO rods are wider than JO (0.27 ± 0.06 µm), and of about the same length (8.3 ± 0.83 µm) (Tables1, S1). They are situated longitudinally, two of them paired, forming a triangle around CONL cilium (Fig. 1 I,J,K). A band of darker material surrounds CONL cilium just proximal of its dilation, filling the space between it, CONS cilium and a triangle of rods (Fig. 1J). Same material is found surrounding two cilia at their base (Figs. 1K, 2A). Rods extend distally into the channel formed by AC (Fig. 3B, Video 5).

Nuclei lie on the outer sides of SC, 7.6 ± 0.42 µm^3^ in volume, occupying 0.35 ± 0.1 of cell bodies; they are larger than SC nuclei in JO. There are 4.2 ± 1.71 mitochondria in SC, altogether 0.75 ± 0.18 µm^3^ in volume (Tables 2, S2, S3).

AC surrounds the triangle of scolopale rods with CONL cilium in center, forming a thin channel, walls of which attach to the rods by intercellular junctions. Distally, rods terminate and the granular material of lumen is replaced by a cylindrical cap (Figs. 1H, 2A) that encloses the cilium tip, which protrudes a bit further. Junctions attach the cap to the AC; they are associated with dense microtubule bundles that extend to the distal end of AC (Figs. 1G, 2A,E). Elongated tips of AC are immersed into the indentations on the circular rim of anellus, proximal of JONL cilia tips (Fig. 2E, Videos 2, 3).

In CO, AC bodies’ volume (20.2 ± 2.0 µm^3^) and nuclei volume (8.2 ± 0.22 µm^3^) are bigger than in JO (Tables 2, S2, S3). Nuclei occupy 0.40 ± 0.05 of AC. AC contain 4.2 ± 2.6 mitochondria per cell, which are the least voluminous in CO (0.31 ± 0.04 µm^3^) (Tables 2, S2, S3). Large vesicles with electron-dense material are found in the distal part of AC.

Structure and dimensions of CO and JO in the second specimen were identical to the first specimen (Table S4).

### JO and CO comparative morphological analysis

#### Structure of the pedicel-flagellum joint

In contrast with shallow depressions of joint membrane observed in *M. viggianii,* depressions in larger insects are usually large and elongated and anchor JO scolopidia^12,19^. A circular apodeme of the pedicel was found in some species, though prongs on it were not described previously; it seems to be the anchor of suspension fibers and joint membrane^20,21^.

The points of CO scolopidia attachment are variable, namely the lateral cuticle of the pedicel^22^, cuticle of the third segment^23^, or direct attachment to the flagellar base^24^, like the one in *M. viggianii*. Sometimes, CO is absent altogether^25^.

#### Scolopidia arrangement and number

**Figure 6:**
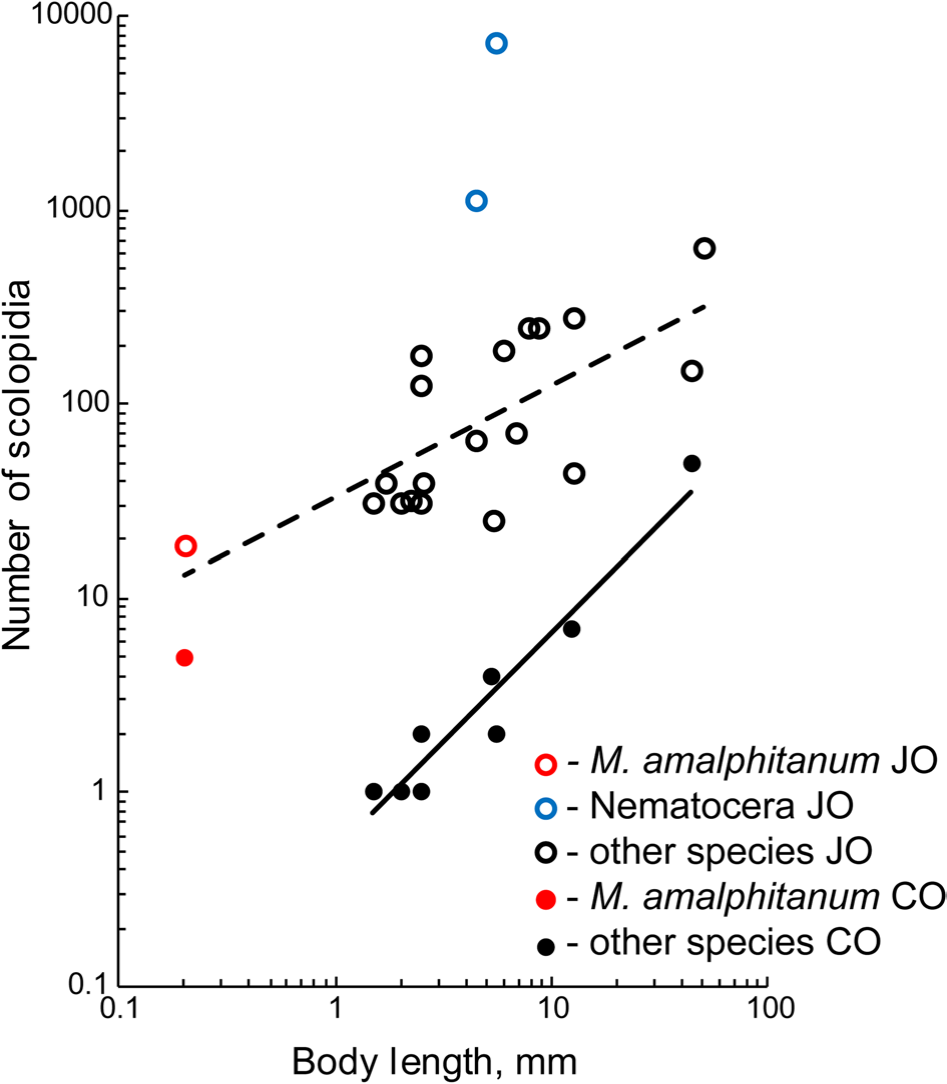
Changes in number of JO and CO scolopidia with body size in insects. For this chart the OLS method was used; the dashed line designates JO OLS, the solid line designates CO OLS. Data source see Table S7.

The circular arrangement of JO with the internal position of CO is typical in insects. *M. viggianii* has the minimum number of JO scolopidia among the studied insects, 1.3–382 times less than in larger species. We analyzed the available data and found that, with the massive JO of Nematocera excluded, a strong correlation (p < 0.01) is observed between the JO scolopidia number and the body size (Fig. 6, Table S5).

Surprisingly, most of the studied species have less CO scolopidia then *M. viggianii*. It is reduced to only 1–2 scolopidia in Diptera and Thysanoptera^21,26^, and sometimes is absent altogether^25^. CO scolopidia number is correlated with the body size in insects, but *M. viggianii* CO does not fit within this trend (Fig. 6, Table S5). The functions of CO are unclear and seem to be variable due to the different points of attachment of scolopidia and their number.

#### Accessory cells

Seven subepidermal accessory cells in *M. viggianii* are divided in two distinct groups—five proximal, which anchor the scolopidia of CO, and two distal (Video 1). It is possible that the five proximal accessory cells of *M. viggianii* are in fact the accessory cells of five CO scolopidia, though one of them is reduced and does not fulfill its purpose as a ligament. In JO of *M. viggianii* there are no accessory cells, though they were found in two other Hymenoptera species, *Neodiprion sertifer* and *Camponotus vagus*^19,27^. Studies on Blattodea, Hemiptera and Thysanoptera suggest an evolutionary trend of independent reduction of accessory cells in several different groups of insects^21,28,29^.

#### Sensory neurons

In chordotonal organs, two types of sensory neurons are often recognized. Type 1 neurons have cilia with a regular axoneme and a dilation with an electron-dense core; the axoneme is restored above the dilation. In type 2 neurons, cilia lose their axoneme distally, forming a dilation filled with numerous microtubules. The most common configuration in JO is 2 shorter type 1 cilia with thick roots, and 1 longer type 2 cilium with a reduced root; in CO usually 2 cilia of type 1 are observed^30^. In *M. viggianii*, CONL is a type 1 cilium, but other cilia are not typical. We presume that JONS and CONS are type 1 cilia that lost their dilations, and JONL is a highly reduced type 2 cilium. Similar deviations were reported for larger Hymenoptera^12,19^.

A number of other features found in *M. viggianii* JO are unique or rare. The absence of proximal basal body was never observed in chordotonal organs, though single basal bodies are sometimes found in other insect mechanoreceptors, for example, campaniform sensilla^31^. Intercellular junctions between JO cilia are rare, reported only in *Thrips validus* Uzel, 1895 (Thysanoptera: Thripidae) and *C. vagus*^21,27^. The sharp turn of the distal basal body, which results in the bending of cilia in JO, was observed in a few species^28,32^. Interestingly, there are no observations of IDS bulbs in insects’ JO and CO, like the ones found in *M. viggianii,* though vibration-sensitive chordotonal organs are often characterized by the IDS bulb^30^. They provide additional space for mitochondria and expand the surface of dendritic tip, but the functional advantages of such features are obscure. Lengths of IDS (6.7 μm) and cilia (6.6 μm) in JO of *M. viggianii* fit within the diversity of the larger insects (0.7–22.5 μm and 6.6–64 μm, respectively). Cilia diameters vary from 0.23 μm to 0.30 μm in JO and CO both, with cilia of *M. viggianii* being 0.30 μm in diameter. In dilations cilia are approximately twice as wide.

#### Scolopale cells

SC have a reticulum of vacuoles proximally, the so-called “labyrinth,” which supposedly secrets the ion regulation material^30^. Its absence in *M. viggianii* is extremely unusual, reported only for *Periplaneta americana* CO^28^. Despite the common conception that scolopale lumen is tightly sealed^33^, in *M. viggianii* JO we found that lumen is connected to the distal cavity, formed by two accessory cells and filled with the same granular material as lumen (Fig. 2E). It could be possible that SC labyrinths were reduced after fulfilling their secretory purpose to provide space in the miniature pedicel, while the secreted material was stored in the cavities and lumens, acting as ion regulator. Discrete bands of darker material, that we found in CO, were also observed in *P. americana* CO^6^. These bands should restrict cilia from the deformation proximally, limiting the strain applied by the flagellum movements to the cilia tips, where mechanosensitive channels are supposedly situated^34^.

There are 6–12 scolopale rods in the SC of the JO and 5–12 in that of the CO in larger insects, while *M. viggianii* has only 4 rods in the SC of both organs. Scolopale rods are either organized in a cylindrical cage that fuses distally^35^, or form a continuous cylinder^19^. Open mesh structure that we described in *M. viggianii* JO is unique in insects. In 2 species of collembola, rods that form a semi-circle or occur only on one side of lumen were found in SC of external sensilla, even though rods in collembolan chordotonal organs acquire typical formation^36^. The proposed functions of rods are reinforcement of the lumen and application of a straightening force along the length of the cilia^32^; their degenerate structure in *M. viggianii* JO does not seem sufficient for these purposes. However, they still anchor bases of JONS cilia, whose tips are in turn connected with JONL cilia by junctions. Thus, stretching of scolopidia by flagellum movement should lead to the angle change of the bent area of JONS cilia base, where mechanosensitive channels could reside^32^. Absence of tubular body and multiple microtubules typical for JONL supports this speculation, as they can not act as cytoskeleton for mechano-electrical transduction in *M. viggianii* JO. Fuzzy desmosomes between the JONS of *M. viggianii* and scolopale rods, called non-laminated collar, were also reported for *P. americana* and *A. aegypti*^26,28^.

In larger insects, SC nuclei were observed proximally; like in *M. viggianii*, their SC are deficient in cytoplasm and mitochondria^37^. The peculiar elongated and twisted shape of SC nuclei and variations in their positions in *M. viggianii* JO could result from the lack of space in a miniaturized pedicel.

SC secretes sheath in JO and and a cup, a sheath variation, in CO^38^. The conic CO cap with a distal opening is unusual for antennal chordotonal organs. Such caps were found in Ephemeroptera unique mononematic JO, but JONL cilia tips did not penetrate them^25^. Cilia tips that protrude further, outside the cap like in *M. viggianii*, were described in the subgenual organ of *Chrysoperla carnea*^39^. CO caps in *M. viggianii* are the shortest (2.0 μm) among the studied insects (6.4–24.0 μm), while their diameter (0.70 μm) fits within the observed variability (0.55–2.7 μm).

#### Attachment cells

AC usually contain numerous microtubules that often form bundles^25^. In CO they are sometimes associated with desmosomes between the AC and the cup^24^. In JO they in rare cases acquire rod-like structure, like the one observed in *M. viggianii*^19^. AC nuclei are often flattened and elongated, and for them abundant mitochondria were reported, unlike *M. viggianii* AC^29^.

#### Volumetric analysis

We compared volumetric data with the data on lamina and medulla interneurons (LI and MI, respectively) of *Trichogramma brassicae* Bezdenko, 1968 (Hymenoptera: Trichogrammatidae) and photoreceptors of *Trichogramma evanescens* Westwood, 1833 (Hymenoptera: Trichogrammatidae)^40,41^. These two species have body 1.5–2 times longer than *M. viggianii*. It would seem that photoreceptors are the most voluminous (20.4 ± 6.0 µm^3^), while JO and CO neurons are smaller, and interneurons have the least cell body volume (6.7 ± 1.0 µm^3^ in LI, 3.8 ± 0.63 µm^3^ in MI) (Table S6). Photoreceptors and CO contain larger volumes of mitochondria (1.9 ± 0.69 µm^3^ and 1.5 ± 0.77 µm^3^) than JO and LI (0.56 ± 0.38 µm^3^ and 0.53 ± 0.18 µm^3^), while MI have the least mitochondria by volume (0.15 ± 0.05 µm^3^). Since the higher mitochondria volume implies higher energetic requirements of the cell, an assumption can be made that JO and interneurons possess lower metabolic activity than photoreceptors and CO. Nuclei of JO and CO neurons of *M. viggianii* are approximately 3 times larger than nuclei of *Trichogramma* species’ interneurons and photoreceptors. This could possibly be explained by the fact that *M. viggianii* have one of the largest genome size in Chalcidoidea^42^. It could be also possible that in *M. viggianii* chromatin is less compact and occupies more volume. Nuclei occupy about 73% of neuron cell bodies in *M. viggianii* JO, in CO and interneurons the ratio is around 50%, and only 13% in photoreceptors^40,41^.

We investigated whether cell body volumes depend on the location of cells in JO and CO. Bubble charts of JO and CO neurons’, AC and SC volumes were made with relation to the cells’ position in the pedicel, but no correlation was observed (Figure S1).

### Miniaturization and optimization of *M. viggianii* JO and CO

*M. viggianii* JO contains only 19 scolopidia, which is the smallest amount among insects. A few structures are significantly reduced: *M. viggianii* has the shortest CO caps, the thinnest articular cuticle and fibers and the smallest number of scolopale rods in JO and CO. Its JO accessory cells are reduced. An unusual absence of SC labyrinths and the elongation and warping of SC nuclei may be linked to miniaturization as well.

In larger insects, JO and CO are often variable in scolopidia organization. Scolopidia of one specimen can differ in number of sensory neurons^23,24^, and in Nematocera scolopidia form several distinct morphological groups^26^. The number of cilia reaching the articular membrane is variable in *C. vagus*^27^; in aphids, an intraspecies variation in number of CO scolopidia was found^23^. Such inconsistency was not observed in *M. viggianii*. All of its JO and CO scolopidia are of identical composition in both studied specimen. This lack of variation implies a high level of optimization and is likely a miniaturization-related adaptation, as the same phenomena was observed in the external antennal sensilla of *M. viggianii* ^18^.

#### Unique features of M. viggianii JO and CO

Pedicel chordotonal organs of *M. viggianii* possess numerous distinctive traits, mostly concerning various supportive structures. Unique features of extracellular structures are the conic shape of CO cap with a distal opening and a protruding cilia tip, prongs on the circular apodeme and uneven thickening of the fibers that attach to them. Within cells, remarkable organization of supportive structures was found in JO. JONL cilia have extremely reduced axoneme and lack distal basal body, while rods of SC acquire unique open mesh structure. Some of them are anchored in dendritic bulbs, which were not observed in larger insects’ JO and CO. The distal cavity filled with granular material and connected to the lumens of JO scolopidia was not described in larger insects, whose lumens are presumed to be tightly sealed.

#### Estimation of limits to JO and CO miniaturization

Comparing the available data, we found that the dimensions of pedicel correlate with the body size in insects. However, *M. viggianii* has a pedicel much longer and wider than it should have to fit within this trend. The same phenomenon is observed in case of *M. viggianii* CO scolopidia number (Fig. 6, Table S5). It is higher than in most examined species, in which it correlates with the body size (Table S5). It could be possible that further reduction of pedicel dimensions and CO scolopidia number would considerably affect the JO and CO functionality, and because of this stronger miniaturization is not observed.

The nuclear-cytoplasmic ratio is considered one of the limiting factors of neuron miniaturization^43^. Studies based on two-dimensional images evaluated the limit of 90% in neurons^44^, but this estimation was revised and a new limit of about 50% was proposed based on volumetric studies^40,41^. In *M. viggianii*, JONS exceed this limit, having a ratio of 71%; in JONL, it is even higher (77%), setting a new limit of nuclear-cytoplasmic ratio in neurons.

#### Scaling of sense organs in parasitoid wasps

A drastic reduction of the number of organ subunits as the one observed in JO is a common trend in sense organs miniaturization. In three *Megaphragma* species, number of antennal sensilla was found to be 3–225 times smaller than in larger parasitoid wasps^18^. In insect compound eyes, number of ommatidia was found to dramatically decrease with the reduction of the body size in Hymenoptera^45^, Lepidoptera^46^ and Coleoptera^47^.

Minute sensory organs demonstrate a lack of variation in their structure due to a high level of optimization, as observed in JO and CO of *M. viggianii*. It was found that antennal sensilla of three *Megaphragma* species are invariable in number, distribution and position within species^18^. Another miniaturization-related trait is the optimization of forms and placement of cell nuclei, as in SC of JO in *M. viggianii,* which allows using space more efficiently. Such reorganization of nuclei was observed in photoreceptor cells of *M. mymaripenne* and *T. evanescens*, and sensory cells of the latter species^41,48^.

Despite the size-related adaptations described above, overall complexity of JO and CO is retained in *M. viggianii*, and their gross structure plan is similar to that of larger insects. The same pattern was observed in the antennal sensilla of *Megaphragma*, which demonstrated morphology and diversity of sensilla types similar to larger parasitoid wasps^18^. Ultrastructure of *M. mymaripenne* ommatidia was found to be comparable to such of larger *T. evanescens*^48^. These observations imply that the complex structure of sense organs is hard to optimize on a small scale without losing its sensitivity, which is crucial for the fitness of miniaturized organisms.

## Methods

### Material

Adult female *Megaphragma viggianii* Fusu, Polaszek and Polilov 2022 (Hymenoptera: Trichogrammatidae) were reared from eggs of *Heliothrips haemorrhoidalis (*Bouché, 1833) (Thysanoptera: Thripidae). Detailed protocol can be found in previous publication^49^. The head was dissected from the body in the cold fixative and immediately thereafter transferred to fresh fixative of 4 °C for 1 h, which consisted of 1% glutaraldehyde (GA) and 1% osmium tetroxide (OsO_4_) in 0.1 M sodium cacodylate buffer (pH = 7.2). The material was then washed in the same buffer and fixed for 2 h in 2% GA in the buffer at 4 °C. Next, the material was washed in the buffer and post-fixed for 16 h in 2% OsO_4_ in the buffer at 4 °C. After fixation material was washed with double distillate water, and then subjected to a 1% UA solution in ddH_2_O overnight at 4 °C, and then placed (in the same solution) into a constant-temperature oven for 2 h at 50 °C. The specimens were then washed in ddH_2_O and contrasted with Walton’s lead aspartate solution (2 h, 50 °C). Material was then washed in ddH_2_O. Subsequently, dehydration of the material was continued using ethanol and acetone. The material was then placed in a mixture of an embedding medium (Epon, Sigma) and acetone (1:2) for 2 h at RT, and then in 1:1 mixture overnight at RT, after which the samples were transferred to a pouring medium for 5 h at RT. The samples were ultimately transferred to silicone embedding molds with fresh Epon and placed in a constant-temperature oven for 48 h at 60 °C.

### FIB-SEM imaging

The specimens were studied using custom FIB-SEM (Zeiss Merlin scanning electron microscope that has a Zeiss Capella focused ion beam)^50^. To image an entire head we used 2 MHz pixel rates with a 2nA primary electron beam with final voxels that were sampling at 8 × 8 nm in x and y and milled with effective 8 nm increments. Image stacks of two specimens were acquired. The full pedicel of one specimen was used for the three-dimensional (3D) reconstruction**,** volumetric analysis and morphometry. In the second specimen, a part of the pedicel was damaged, but we were still able to partly use it for morphometry and interspecimen comparison.

### 3D reconstruction

3D reconstruction was carried out with Bitplane Imaris 9.5. The volumes of cells and cell structures were calculated based on the 3D models using the Imaris statistics module. Volumes of sensory neurons cell bodies were calculated without cell processes.

### Morphometry

All dimensions were measured on the FIB-SEM images using measurement tools of Bitplane Imaris. Normality test, descriptive statistic, the Mann–Whitney U test and SMA were performed in R software using the package *smatr* for SMA^51^.

## Supplementary Information

Supplementary Information 1.

Supplementary Video 1.

Supplementary Video 2.

Supplementary Video 3.

Supplementary Video 4.

Supplementary Video 5.

## Data Availability

The datasets generated and/or analyzed during the current study are available in the supplementary information files. Original electron microscope images available from the corresponding author on reasonable request.
