# Supplementary material for: The 3D ultrastructure of the chordotonal organs in the antenna of a microwasp remains complex although simplified
Source: Sci Rep. 2022 Nov 23;12:20172. doi: 10.1038/s41598-022-24390-4 (PMC9691716; doi:10.1038/s41598-022-24390-4)
Supplement: Supplementary file 1 — Supplementary Information 1. [file 41598_2022_24390_MOESM1_ESM.pdf]

The 3D ultrastructure of the chordotonal organs in the antenna of a microwasp remains complex although simplified

Anna V. Diakova[1]*, Anastasia A. Makarova[1], Song Pang[2,3], C. Shan Xu[2,4], Harald Hess[2], Alexey A. Polilov[1]*

[1] Department of Entomology, Faculty of Biology, Lomonosov Moscow State University, Moscow, Russia

[2] Janelia Research Campus of the Howard Hughes Medical Institute, Ashburn, United States

[3] Yale School of Medicine, New Haven, CT, USA

[4] Department of Cellular and Molecular Physiology, Yale School of Medicine, New Haven, CT, USA

* Correspondence to polilov@gmail.com and anndiakova@yandex.ru

Supplementary materials:

Figure S1. Bubble charts of volumes of cells of *M. viggianii* JO and CO with relation to the position in the pedicel.

Table S1. Significance of the differences among group means of cell and cellular structures dimensions of female *M. viggianii* chordotonal organs calculated with ANOVA.

Table S2. Significance of the differences among group means between JO and CO of *M. viggianii* cell and organelles volumes calculated with ANOVA.

Table S3. Significance of the differences among group means of cell and organelles volumes of female *M. viggianii* chordotonal organs calculated with ANOVA.

Table S4. Cell and cellular structures dimensions of female *M. viggianii* chordotonal organs, raw data.

Table S5. P-values, slope p-values and R^2 of regression analyses performed on the data on the number of JO and CO scolopidia, pedicel length and diameter in insects.

Table S6. Cell and organelle volumes and nuclei volume to cell volume ratios of *M. viggianii* chordotonal organs and *Trichogramma* lamina and medulla interneurons and photoreceptors.

Table S7. References for the data used in regression analyses.

Table S8. Organelle volumes (μm^3), number of mitochondria and nuclei volume to cell volume ratios of female *M. viggianii* chordotonal organs, raw data.

Table S9. Cell volumes (μm^3) in female *M. viggianii* chordotonal organs, raw data.

Video 1. 3D reconstruction of the ultrastructure of *Megaphragma viggianii* pedicel.

Video 2. 3D reconstruction of *Megaphragma viggianii* pedicel-flagellum joint.

Video 3. 3D reconstruction of the ultrastructure of *Megaphragma viggianii* pedicel with emphasis on the pedicel-flagellum joint region.

Video 4. 3D reconstruction of the ultrastructure of *Megaphragma viggianii* Johnston's organ scolopidium.

Video 5. 3D reconstruction of the ultrastructure of *Megaphragma viggianii* central organ scolopidium.

Figure S1. Bubble charts of volumes of cells of *M. viggianii* JO and CO with relation to the position in the pedicel: (A) AC; (B) SC; (C) JONL, CONL; (D) $JONS_1$, CONS; (E) $JONS_2$.

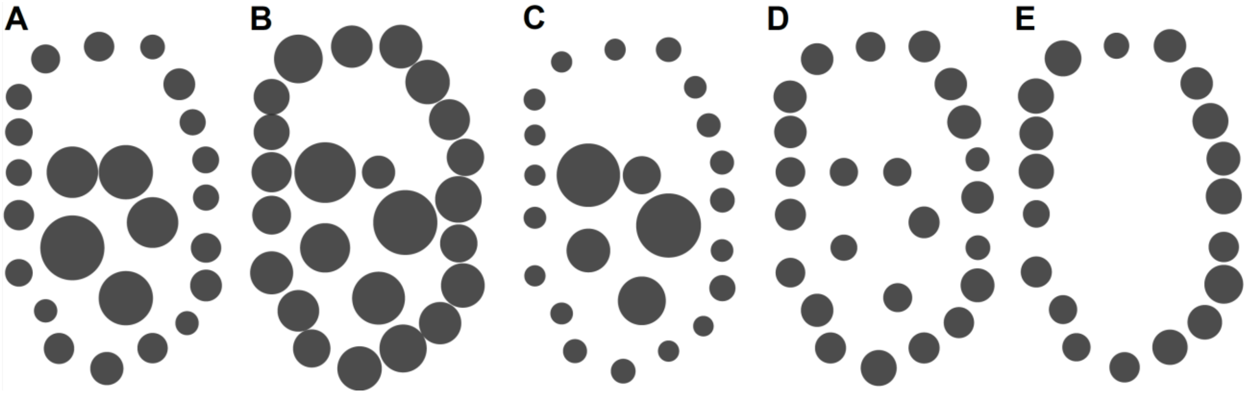

Table S1. Significance of the differences among group means of cell and cellular structures dimensions of female *M. viggianii* chordotonal organs calculated with ANOVA. Asterisks (*) indicate significant difference (p-value < 0.05).

Cell type (sample size)	Length	Diameter
JO/CO scolopidia (10/6)	0.00136*	0.870
JO/CO scolopale rods (10/10)	0.9397	0.0340*
JONL/JONS IDS (10/10)	0.000181*	0.000180
JONL/JONS cilia (10/10)	0.0961	0.136
CONL/CONS IDS (6/6)	0.575	0.00499*
CONL/CONS cilia (6/6)	0.00507*	0.725
JONL/CONL IDS (10/6)	0.531	0.00135*
JONL/CONS IDS (10/6)	0.531	0.0255*
JONS/CONL IDS (10/6)	0.0000869*	0.128
JONS/CONS IDS (10/6)	0.0000869*	0.00136*
JO neurons cilia/CONL cilia (20/6)	0.000292*	0.545
JO neurons cilia/CONS cilia (20/6)	0.0105*	0.545

Table S2. Significance of the differences among group means between JO and CO of *M. viggianii* cell and organelles volumes calculated with ANOVA. Asterisks (*) indicate significant difference (p-value < 0.05).

**Cell type (sample size)**	**Nuclei volumes**	**Mitochondria volumes**	**Mitochondria number**	**Nuclei volume to cell volume ratio**
JO neurons/CO neurons (15/8)	0.512	0.00484*	0.00111*	0.000333*
JO SC/CO SC (5/4)	0.0303*	0.0303*	0.465	0.665
JO AC/CO AC (5/4)	0.0303*	0.0303*	0.0530	0.0303*
JONS/CONS (10/4)	0.00847*	0.444	–	–
JONS/CO neurons (10/8)	–	–	0.00590*	–
JONL/CONS (5/4)	0.885	0.0294*	–	–
JONL/CO neurons (5/8)			0.00802*	–
JONS/CONL (10/4)	0.00847*	0.00847*	–	–
JONL/CONL (5/4)	0.0303*	0.0294*	–	–

Table S3. Significance of the differences among group means of cell and organelles volumes of female *M. viggianii* chordotonal organs calculated with ANOVA. Mitochondria volumes: A, C, upper half of the table; nuclei volumes: A, C, bottom half of the table; nuclei volume to cell volume ratios: B, D, upper half of the table; number of mitochondria: B, D, bottom half of the table. Asterisks (*) indicate significant difference (p-value < 0.05). Sample sizes – cell volumes: JONL – 19, JONS – 38, JO SC – 19, JO AC – 19, CONL – 5, CONS – 5, CO AC – 5, CO SC – 5; organelles volumes: JONL – 5, JONS – 10, JO SC – 5, JO AC – 5, CONL – 4, CONS – 4, CO AC – 4, CO SC – 4.

**A**	JONS	JONL	JO SC	JO AC	**B**	JONS	JONL	JO SC	JO AC
JONS		0.00836*	0.00847*	0.00847*	JONS		0.148	0.00847*	0.00847*
JONL	0.0337*		0.467	0.884	JONL	0.0121*		0.0303*	0.0303*
JO SC	0.00847*	0.312		0.312	JO SC	0.0843	1		0.0303*
JO AC	0.00847*	0.112	0.112		JO AC	0.0076*	0.0863	0.288	
**C**	CONS	CONL	CO SC	CO AC	**D**	CONS	CONL	CO SC	CO AC
CONS		0.0303*	0.312	0.0303*	CONS		0.193	0.0303*	0.0303*
CONL	0.0303*		0.0303*	0.0303*	CONL	0.1886		0.112	0.193
CO SC	0.245	0.0303*		0.0304*	CO SC	0.0420*	0.0303*		0.306
CO AC	0.0303*	0.0303*	0.0606		CO AC	0.146	0.0606	0.883	

Table S4. Dimensions of cells and cell structures dimensions in the chordotonal organs of female *M. viggianii*, raw data, µm, L – length, D – diameter.

**Specimen**	1	1	1	1	1	2	2	2	2	2
JO scolopidium L	16.0	15.0	16.0	14.2	16.1	15.0	14.5	15.52	16.8	16.5
JO scolopidium D	4.53	4.72	3.94	2.79	2.45	5.76	3.66	2.91	3.30	3.15
CO scolopidium L	21.0	20.5	22.0	24.5	21.8	21.1	-	-	-	-
CO scolopidium D	4.10	3.49	3.34	2.55	4.05	3.96	-	-	-	-
JO scolopale rod L	7.95	8.21	8.64	7.80	8.10	8.20	7.98	8.14	8.05	7.9
JO scolopale rod D	0.55	0.28	0.23	0.29	0.35	0.47	0.57	0.34	0.28	0.43
CO scolopale rod L	9.24	8.16	8.52	9.85	9.05	7.87	7.42	7.60	7.48	7.90
CO scolopale rod D	0.26	0.34	0.19	0.25	0.17	0.30	0.36	0.22	0.32	0.26
JONL IDS L	8.00	8.70	9.70	8.87	8.60	8.22	8.57	9.98	9.50	8.60
JONL IDS D	0.47	0.31	0.29	0.35	0.35	0.42	0.34	0.39	0.34	0.38
JONS IDS L	5.20	4.40	4.18	4.30	3.47	6.20	3.90	3.30	5.10	4.20
JONS IDS D	1.26	1.07	0.86	0.83	0.68	0.63	0.60	0.67	1.04	0.75
JONL cilium L	6.77	6.15	7.09	6.55	6.70	6.78	7.08	6.22	7.19	7.26
JONL cilium D	0.32	0.28	0.32	0.31	0.28	0.34	0.32	0.35	0.3	0.29
JONS cilium L	5.87	6.40	6.80	6.52	6.74	6.10	6.30	6.20	6.55	7.17
JONS cilium D	0.31	0.27	0.28	0.30	0.27	0.28	0.33	0.28	0.34	0.29
CONL IDS L	4.50	2.76	7.80	5.12	4.31	5.18	–	–	–	–
CONL IDS D	0.86	0.74	0.58	0.66	0.67	0.60	–	–	–	–
CONS IDS L	6.00	4.66	4.87	4.24	3.41	3.66	–	–	–	–
CONS IDS D	0.56	0.38	0.38	0.53	0.41	0.55	–	–	–	–
CONL cilium L	11.5	9.9	10.0	10.8	10.6	9.67	–	–	–	–
CONL cilium D	0.31	0.27	0.29	0.28	0.31	0.31	0.32	0.27	0.28	0.31
CONS cilium L	6.05	5.93	5.88	5.41	5.52	6.8				
CONS cilium D	0.33	0.28	0.28	0.28	0.31	0.31	0.31	0.30	0.30	0.30
cilium dilation diameter	0.52	0.52	0.62	0.58	0.53	0.64	0.64	0.58	0.66	0.61
cup L	2.05	2.07	2.05	2.15	1.75	2.10	2.25	1.80	1.66	2.15
cup D	0.65	0.66	0.54	0.57	0.63	0.65	0.69	0.73	0.70	0.67

Table S5. P-values, slope p-values and R^2 of regression analyses performed on the data on the number (N) of JO and CO scolopidia, pedicel length (L) and diameter (D) in insects. Asterisks (*) indicate significant difference (p-value < 0.01). Slope p-value (0) shows if the slope of SMA regression is significantly different from 0, slope p-value (1) shows if the slope of SMA regression is significantly different from 1. The references for the data are given in Table S7.

	**p-value**	**R^2**	**Slope**	**Slope p-value (0)**	**Slope p-value (1)**
N of JO scolopidia	0.000703*	0.500	0.813	2.22e-16*	0.242
N of CO scolopidia	2.09e-5*	0.907	1.18	< 2.22e-16*	–
Pedicel L	1.53e-6*	0.887	0.787	< 2.22e-16*	–
Pedicel D	0.000214*	0.797	0.996	1	0.982

Table S6. Cell and organelle volumes and nuclei volume to cell volume ratios of *M. viggianii* chordotonal organs and *Trichogramma* lamina and medulla interneurons (LI, MI) and photoreceptors; mean ± sd, μm^3. *Trichogramma* data from sources 40 and 41.

	**Cell volume**	**Nuclei volume**	**Nuclei volume to cell volume ratio**	**Mitochondria volume**
M. viggianii photoreceptors	26.5±10.2	7.4±1.1	0.31±0.07	1.7±0.88
M. viggianii CO neurons	15.7±5.7	9.0±2.3	0.53±0.08	1.5±0.77
M. viggianii JO neurons	11.3±2.1	7.5±0.81	0.73±0.06	0.56±0.38
Trichogramma photoreceptors	20.4±6.0	2.4±0.27	0.13±0.04	1.9±0.69
Trichogramma LI	6.7±1.0	2.8±0.26	0.43±0.04	0.53±0.18
Trichogramma MI	3.8±0.63	1.8±0.25	0.48±0.05	0.15±0.05

Table S7. References for the data used in regression analyses.

Reference	Species
Ai, Nishino, & Itoh, 2007, Tsujiuchi et al., 2007	*Apis mellifera* Linnaeus, 1758 (Hymenoptera: Apidae)
Bode, 1986	*Thrips validus* Uzel, 1895 (Thysanoptera:Thripidae)
Bode, 1986	*Aeolothrips intermedius* Bagnall, 1934 (Thysanoptera:Aeolothripidae)
Bode, 1986	*Haplothrips aculeatus* Fabricius, 1803 (Thysanoptera: Phlaeothripidae)
Bromley et al., 1980	*Aphis pomi* de Geer, 1773 (Hemiptera: Aphididae)
Bromley et al., 1980	*Macrosiphum euphorbiae* Thomas, 1878 (Hemiptera:Aphididae)
Bromley et al., 1980	*Pemphigus bursarius* Linnaeus, 1758 (Hemiptera: Aphididae)
Fedorova & Zhantiev, 2009	*Fleuria lacustris* Kieffer, 1924 (Diptera: Chironomidae)
Fedorova & Zhantiev, 2009	*Chironimus plumosus* Linnaeus, 1758 (Diptera: Chironomidae)
Fedorova & Zhantiev, 2009	*Dasyhelea versicolor* Winnertz, 1852 (Diptera: Ceratopogonidae)
Hallberg, 1981	*Neodiprion sertifer* Geoffroy 1785 (Hymenoptera: Diprionidae)
Howse & Claridge, 1970	*Oncopsis flavicollis* Linnaeus, 1761 (Hemiptera: Cicadellidae)
Jeram & Pabst, 1996	*Nezara viridula* Linnaeus, 1758 (Heteroptera: Pentatomidae)
K. S. Boo & Davies, 1980	*Simulium vittatum* Latreille, 1802 (Diptera: Simuliidae)
Kyung Saeng Boo & Richards, 1975), Belton, 1989, Fedorova & Zhantiev, 2009	*Aedes aegypti* Linnaeus, 1762 (Diptera: Culicidae)
Rossi Stacconi & Romani, 2013	*Hyalesthes obsoletus* Signoret 1865 (Hemiptera: Cixiidae)
Rossi Stacconi & Romani, 2013	*Metcalfa pruinosa* Say, 1830 (Hemiptera: Flatidae)
Rossi Stacconi & Romani, 2013	*Scaphoideus titanus* Ball, 1932 (Hemiptera: Cicadellidae)
Schmidt, 1974	*Cloeon dipterum* Linnaeus, 1761 (Ephemeroptera: Baetidae)
Toh, 1981, Toh & Yokohari, 1985	*Periplaneta americana* Linnaeus, 1758 (Blattodea: Blattidae)
Vande Berg, 1971	*Manduca sexta* Linnaeus, 1763 (Lepidoptera: Sphingidae)

Table S8. Organelle volumes (µm^3), number of mitochondria and nuclei volume to cell volume ratios of female *M. viggianii* chordotonal organs, raw data.

Scolopidium	Cell	Nuclei volume	Mitochondria volume	Number of mitochondria	Nuclei volume to cell volume ratio
JO 1	JONS	8.02	0.98	3	0.69
JO 1	JONS	7.34	0.80	4	0.72
JO 1	JONL	6.47	0.14	3	0.76
JO 1	SC	6.21	0.42	2	0.31
JO 1	AC	6.07	0.17	2	0.56
JO 2	JONS	8.18	0.76	5	0.66
JO 2	JONS	8.21	0.54	5	0.75
JO 2	JONL	7.62	0.07	2	0.82
JO 2	SC	6.29	0.44	6	0.35
JO 2	AC	5.98	0.02	1	0.53
JO 3	JONS	7.55	0.49	4	0.78
JO 3	JONS	9.00	0.90	4	0.75
JO 3	JONL	7.16	0.11	2	0.80
JO 3	SC	6.88	0.12	1	0.39
JO 3	AC	5.79	0.13	2	0.53
JO 4	JONS	7.77	1.15	8	0.63
JO 4	JONS	7.35	0.63	7	0.69
JO 4	JONL	5.94	0.11	3	0.68
JO 4	SC	5.82	0.06	3	0.32
JO 4	AC	5.62	0.03	1	0.51
CO 1	CONL	9.23	1.64	14	0.46
CO 1	CONS	7.05	1.02	8	0.51
CO 1	SC	7.74	0.72	5	0.49
CO 1	AC	8.21	0.36	8	0.41
CO 2	CONL	12.9	3.01	22	0.40
CO 2	CONS	7.02	0.59	6	0.54
CO 2	SC	8.02	0.54	2	0.28
CO 2	AC	8.31	0.27	3	0.44
CO 3	CONS	7.12	0.88	7	0.58
CO 3	CONL	10.3	1.98	7	0.50
CO 3	SC	7.53	0.98	4	0.32
CO 3	AC	7.84	0.33	2	0.33
CO 4	CONL	11.3	1.69	12	0.59
CO 4	CONS	7.25	1.09	12	0.64
CO 4	SC	7.02	0.76	6	0.28
CO 4	AC	8.30	0.28	4	0.41

Table S9. Cell volumes (μm^3) in female *M. viggianii* chordotonal organs, raw data.

Scolopidium no.	Cell	Cell volume
CO 1	CONL	20.0
CO 1	CONS	13.8
CO 1	SC	15.6
CO 1	AC	19.8
CO 2	CONL	32.0
CO 2	CONS	13.0
CO 2	SC	28.5
CO 2	AC	18.9
CO 3	CONS	12.3
CO 3	CONL	20.5
CO 3	SC	23.2
CO 3	AC	23.5
CO 4	CONL	23.5
CO 4	CONS	15.2
CO 4	SC	24.8
CO 4	AC	20.0
CO 5	CONL	33.3
CO 5	CONS	14.5
CO 5	SC	30.1
CO 5	AC	18.6
JO 1	JONS	16.8
JO 1	JONS	13.4
JO 1	JONL	12.5
JO 1	SC	19.7
JO 1	AC	10.8
JO 2	JONS	16.0
JO 2	JONS	19.0
JO 2	JONL	10.6
JO 2	SC	22.8
JO 2	AC	10.5
JO 3	JONS	15.8
JO 3	JONS	16.7
JO 3	JONL	11.7
JO 3	SC	17.0
JO 3	AC	9.67
JO 4	JONS	15.7
JO 4	JONS	15.8
JO 4	JONL	10.9
JO 4	SC	16.6
JO 4	AC	10.1
JO 5	JONS	14.8
JO 5	JONS	16.5
JO 5	JONL	10.1
JO 5	SC	18.8
JO 5	AC	9.78
JO 6	JONS	16.0

JO 6	JONS	13.5
JO 6	JONL	9.94
JO 6	SC	18.0
JO 6	AC	10.9
JO 7	JONS	14.5
JO 7	JONS	15.3
JO 7	JONL	10.1
JO 7	SC	20.0
JO 7	AC	10.0
JO 8	JONS	15.4
JO 8	JONS	14.0
JO 8	JONL	10.6
JO 8	SC	19.5
JO 8	AC	8.59
JO 9	JONS	15.0
JO 9	JONS	14.4
JO 9	JONL	10.9
JO 9	SC	17.7
JO 9	AC	11.3
JO 10	JONS	17.7
JO 10	JONS	15.4
JO 10	JONL	11.6
JO 10	SC	20.7
JO 10	AC	12.1
JO 11	JONS	14.4
JO 11	JONS	15.8
JO 11	JONL	10.8
JO 11	SC	22.4
JO 11	AC	11.1
JO 12	JONL	10.6
JO 12	JONS	19.9
JO 12	JONS	15.6
JO 12	SC	19.9
JO 12	AC	8.55
JO 13	JONS	16.2
JO 13	JONS	17.5
JO 13	JONL	11.5
JO 13	SC	20.5
JO 13	AC	11.6
JO 14	JONS	13.4
JO 14	JONS	14.9
JO 14	JONL	10.9
JO 14	SC	17.6
JO 14	AC	10.9
JO 15	JONS	15.4
JO 15	JONS	17.5
JO 15	JONL	10.5
JO 15	SC	21.8

JO 15	AC	9.66
JO 16	JONS	15.9
JO 16	JONS	11.4
JO 16	JONL	10.6
JO 16	SC	17.4
JO 16	AC	9.80
JO 17	JONS	15.9
JO 17	JONS	15.3
JO 17	JONL	10.4
JO 17	SC	19.1
JO 17	AC	9.49
JO 18	JONS	15.0
JO 18	JONS	16.5
JO 18	JONL	10.4
JO 18	SC	21.0
JO 18	AC	11.7
JO 19	JONS	13.9
JO 19	JONS	14.1
JO 19	JONL	11.3
JO 19	SC	20.4
JO 19	AC	9.01
